# Informed consent, duty of disclosure and chiropractic: where are we?

**DOI:** 10.1186/s12998-020-00342-5

**Published:** 2020-11-04

**Authors:** J. Keith Simpson, Stanley Innes

**Affiliations:** Discipline of Psychology, Exercise Science, Counselling and Chiropractic (PESCC), College of Science, Health, Engineering and Education (SHEE), Murdoch University, Murdoch, Australia

**Keywords:** Chiropractic, Informed consent, Duty of disclosure, Negligence

## Abstract

**Background:**

The COVID-19 pandemic has seen the emergence of unsubstantiated claims by vertebral subluxation-based chiropractors that spinal manipulative therapy has a role to play in prevention by enhancing the body’s immune function. We contend that these claims are unprofessional and demonstrate a disturbing lack of insight into the doctrine of informed consent. As such it is timely to review how informed consent has evolved and continues to do so and also to discuss the attendant implications for contemporary health practitioner practice.

We review the origins of informed consent and trace the duty of disclosure and materiality through landmark medical consent cases in four common law (case law) jurisdictions. The duty of disclosure has evolved from a patriarchal exercise to one in which patient autonomy in clinical decision making is paramount. Passing time has seen the duty of disclosure evolve to include non-medical aspects that may influence the delivery of care. We argue that a patient cannot provide valid informed consent for the removal of vertebral subluxation. Further, vertebral subluxation care cannot meet code of conduct standards because it lacks an evidence base and is practitioner-centered.

The uptake of the expanded duty of disclosure has been slow and incomplete by practitioners and regulators. The expanded duty of disclosure has implications, both educative and punitive for regulators, chiropractic educators and professional associations. We discuss how practitioners and regulators can be informed by other sources such as consumer law. For regulators, reviewing and updating informed consent requirements is required. For practitioners it may necessitate disclosure of health status, conflict of interest when recommending “inhouse” products, recency of training after attending continuing professional development, practice patterns, personal interests and disciplinary findings.

**Conclusion:**

Ultimately such matters are informed by the deliberations of the courts. It is our opinion that the duty of a mature profession to critically self-evaluate and respond in the best interests of the patient before these matters arrive in court.

## Background

The COVID-19 pandemic highlights, amongst other things, all that is *professional* and *unprofessional* within the chiropractic profession. First, we discuss the *unprofessional*. By *unprofessional* be mean behaviour below or contrary to the standards expected in a particular profession [1]. Faced with the pandemic, the International Chiropractors Association (ICA) produced 2 reports on chiropractic and the immune system [2, 3]. While the initial report states members should “not advertise in any form the suggestion that chiropractic can cure, treat, prevent, or mitigate COVID-19” [2]p.3 and the Updated Report states “no claims may be asserted that chiropractic is a prophylactic or cure for COVID-19” [emphasis in the original] [3]p.2 they do state there is a growing body of evidence that there is a relationship between the nervous system and the immune system. Although several mechanisms by which the nervous and immune systems might interact have now been recognized, absent from the report is acknowledgement no credible scientific evidence demonstrate a link between vertebral subluxation and immune function.

The updated report states that spinal subluxation and spinal manipulation impacts neurologic function and endocrine, and immune systems are interdependent [3]. The ICA’s website informs that subluxation detection and removal facilitates optimal life expression, health and human potential [4]. The Reports’ inference is obvious: subluxation removal can help combat the COVID-19 pandemic by boosting the immune system. The reports conclude “research to validate the role of doctors of chiropractic in promoting health and vitality by stimulating a healthy immune response is required” [2] p.13, [3] p.19.

Due to the ICA COVID-19 reports, chiropractic associations and chiropractors around the world who either belong to the ICA or align with this ideology, claimed that patients should undergo spinal manipulation to help combat the COVID-19 virus [5–7]. Such irresponsible action places both themselves and the public at risk.

The ICA reports epitomise the prototypical chiropractic defence against charges of practising medicine without a licence:

- Chiropractic is a vitalistic non-therapeutic, non-allopathic, drugless, non-surgical health science [non-therapeutic meaning chiropractors treat no disease];
- Chiropractic is a health care discipline which emphasizes the inherent recuperative power of the body to heal itself without drugs or surgery;
- Subluxations interfere with nerve function; Interference to nerve function causes brain/body communication interference;
- Interference has a negative impact on the nervous system or organ system functioning thereby adversely affecting optimal health;
- Chiropractors detect and remove subluxations;
- Ergo, by removing subluxations, brain body communication and function are optimised enhancing overall health [8].

The problems with this ideology have been discussed elsewhere [9–12]. Suffice it say that at best chiropractic vertebral subluxation (VS) as a cause of disease is a potentially testable hypothesis, at worst it is a dogmatically retained anachronism that does not have any role in the twenty-first century health care system.

The *professional* within chiropractic is demonstrated by the actions of the chiropractic research community in response to the ICA reports. By *professional* we mean having the recognized attributes of a profession [13] upholding fiduciary duties [14] and abiding by the social contract between the profession and society [15]. The chiropractic research community reviewed the ICA reports and found they “provided no valid clinical scientific evidence that chiropractic care can impact the immune system” [16]. Similarly, the World Federation of Chiropractic’s (WFC) examination of chiropractic and immunity found:

There is no credible scientific evidence that chiropractic spinal adjustment/manipulation confers or boosts immunity. Chiropractors should refrain from any communication that suggests spinal adjustment/manipulation may protect patients from contracting COVID-19 or will enhance their recovery. Doing otherwise is potentially dangerous to public health [17].

Professionalism is further demonstrated by collaborative research examining social media claims by chiropractors that chiropractic treatment can prevent or impact COVID-19 [5, 6]. The results demonstrated alarmingly high and widespread misinformation by chiropractors. These researchers urged all chiropractic stakeholders to view the COVID-19 pandemic as a call to action to eliminate the unethical, unsubstantiated and potentially dangerous claims made by chiropractors who practise outside the boundaries of scientific evidence [5, 6]. Similarly, regulatory agencies advise it is vital that health practitioners only provide information about COVID-19 that is scientifically accurate and from authoritative sources, such as a state, territory or Commonwealth health department or the World Health Organization (WHO) [18].

Assertions such as the ICA’s and others illustrate *unprofessional* conduct. They constitute deceptive conduct by misuse of the scientific literature. They breach fiduciary duties, professional conduct codes, and consumer laws. Such conduct is likely to appeal to fear and lead health care consumers into potentially dangerous decisions [19, 20].

We contend that a chiropractor making the claim or implying that chiropractic boosts immunity or any other unsubstantiated claim cannot fulfil the requirements of informed consent. The primary aim of this paper is to examine this and the implications this failure has. This necessitates initially reviewing the expansion of disclosable information within informed consent (IC). Following consideration of these matters, we will assert that it is impossible to fulfil the requirements of IC as a chiropractor promoting subluxation-based care and in doing so, these practitioners are exposed to liability under the doctrine of informed consent.

The evolution of the scope of the duty of disclosure has other implications for chiropractors. We will also discuss the evolving scope of disclosure as it relates to non-clinical matters such as practitioner characteristics and practice patterns, practitioner qualifications and experience, and practitioner financial interests.

### From consent to disclosure of material risks

The doctrine of informed consent in health care is rooted in the English Common Law doctrine of assault and battery. It developed during the twentieth century from three main sources: moral (fiduciary duty), ethical (client autonomy), and legal (defence against trespass, battery and negligence) [21]. Regardless of which model of physician-patient relationship is used, patient autonomy and the provision of consent are central concepts [22]. The basic principle of consent within the context of health care has remained unchanged for centuries: touching without consent is the tort of battery. Battery within health care requires two elements:

1. Did the patient know that the practitioner would perform the procedure?
2. Did the patient authorize the practitioner to perform the procedure?

If either question elicits a ‘no’ answer, battery has occurred. While it is not the case that all medical care requires touching, chiropractic care typically does. Within this context, touching an un-consenting client would be battery even if the touching is considered beneficial [23].

Consent affords the practitioner lawful justification for touching and hence treatment. If consent is not informed, the practitioner can be found negligent. Exceptions to obtaining consent include necessity and the therapeutic exception. Necessity can be argued, for example, where the patient requires treatment urgently but is unconscious or otherwise unable to decide. Therapeutic exception is varyingly called physician discretion, therapeutic nondisclosure or therapeutic privilege. This exception allows the practitioner to withhold relevant information if disclosing the information might create incapacitating emotional distress or violate a patient’s personal, cultural, or other social requirements. However, therapeutic exception cannot be a justification to avoid a patient’s refusal of care thought necessary by the practitioner. Obviously, invoking therapeutic privilege requires significant judgment by the practitioner. Nondisclosure is likely to be detrimental to the provider/patient relationship and does not sit well with the moral duty for truth telling by the practitioner. Importantly, nondisclosure weakens patient autonomy by limiting a patient’s ability to determine what is done with their own body. From a medico-legal perspective, necessity is a defence to battery, necessity and therapeutic exception are defences to negligence [24].

The term informed consent (IC) is a form of shorthand for all aspects of obtaining permission from a patient. In reality IC is about the duty of the practitioner to provide the patient with sufficient accurate information to allow the patient to make informed decisions. Thus, information disclosure is a dominant concern at the very core of the doctrine of informed consent. As IC developed during the twentieth century it evolved from a paternalistic model to one in which patient autonomy in clinical decision making is paramount [25].

IC can be regarded as a meeting of the minds, analogous to a contractual procedure between a competent, comprehending, uncoerced patient and a health care provider (HCP) who must disclose all relevant information including a valid basis for the proposed care. Having understood the disclosed information, the patient voluntarily consents to or rejects the proposed care plan. The HCP undertakes to provide treatment using their skill, knowledge and judgment to achieve a desired result and the client contracts to compensate the HCP for their services. Failure to disclose all relevant information may vitiate consent and may render the HCP liable in negligence. During the past four decades information considered disclosable has expanded to not only include treatment specific information, but also practitioner specific information [23, 26–30].

Laws governing professional conduct, including consent, are set out legislatively and by court decisions. They are administered through governmental institutions and relevant professional bodies. The courts interpret the laws to which individuals must abide. Court interpretations understandably evolve in keeping with societal changes while respecting the fiduciary relationship between the patient and HCP [31, 32]. Because the law governing IC varies across jurisdictions and countries, we examine disclosure requirements as they emerged from landmark twentieth century cases in four major common law countries – the United Kingdom (UK), the United States of America (US), Canada, and Australia. Readers desirous of detailed analysis are directed to leading texts dealing with each jurisdiction: UK [33], US [34, 35], Canada [36], Australia [37] with the definitive analysis and description of the informed consent concept being Faden and Beauchamp’s text [21].

The 1914 decision in the US case of *Schloendorff v Society of New York Hospital* [38] is credited with establishing the concept of consent and patient autonomy within health care. Justice Cardozo wrote:

Every human being of adult years and sound mind has a right to determine what shall be done with his own body; and a surgeon who performs an operation without his patient's consent commits an assault, for which he is liable in damages.

The 2015 UK case of *Montgomery v Lanarkshire Health Board* [39] became the most recent milestone in the evolution of the duty of disclosure and informed consent. It effectively embedded materiality into the doctrine of disclosure. The Court ruled:

The doctor is therefore under a duty to take reasonable care to ensure that the patient is aware of any material risks involved in any recommended treatment, and of any reasonable alternative or variant treatments.

The 101 years between *Schloendorff v Society of New York Hospital* and *Montgomery v Lanarkshire Health Board* have seen the requirements for disclosure in consent progress from a doctor-centered model in which the practitioner determined the course of care to a patient-centered model in which patient autonomy in decision-making is paramount.

The term informed consent emerged in the 1957 in the US case of *Salgo v Leyland* [40]. Justice Bray recognized that written consent was ineffective if the patient did not understand critical information about the proposed procedure. Justice Bray wrote:

A physician violates his duty to his patient and subjects himself to liability if he withholds any facts which are necessary to form the basis of an intelligent consent by the patient to the proposed treatment. …

The physician has such discretion consistent, of course, with the full disclosure of facts necessary to an informed consent.

By the middle of the twentieth century physicians were expected, but not necessarily compelled, to provide the competent patient with sufficient information to facilitate an informed decision. Typically, disclosure was limited to therapeutic information about the purpose, benefits, and potential risks of a health care intervention, and alternatives to the proposed care including doing nothing. The scope of disclosure was judged by the Bolam test which emerged from the 1957 UK case of *Bolam v Friern Hospital Management Committee* [41]. Simply stated the Bolam test considered what the reasonable physician in the same circumstances would disclose (or withhold).

From 1972 onward, the courts recognised the limitations of the reasonable practitioner standard including: impeding patient autonomy; lack of consensus amongst practitioners; unwillingness of practitioners to speak out against their colleagues [42]. This recognition saw the scope of disclosable information expanded to encompass *material risks* which focuses more on the patient’s interests.

Material risks were defined by Waltz and Scheunerman [43] in their influential 1970 paper. They explained the materiality (importance) of a risk may be initially determined by the physician. Once determined by the practitioner, the risk should be disclosed when the patient would consider it important information in their decision making [43]. Waltz and Scheunerman proposed the following reasonable person standard for both the physician and the courts:

A risk is thus material when a reasonable person, in what the physician knows or should know to be the patient's position, would be likely to attach significance to the risk or cluster of risks in deciding whether or not to undergo the proposed therapy [43]. p. 640

Faden and Beauchamp [21] advised that *materiality* or what is important is the legal litmus test used to determine the extent of disclosure under the reasonable person standard. In other words, patient, not the physician, is the arbiter. Landmark cases in the US (*Canterbury v Spence* 1972 [44]), Canada (*Reibl v. Hughes * 1980 [45]), Australia (*Rogers v Whitaker* 1992 [46]) and the UK (*Montgomery v Lanarkshire Health Board* 2015 [39]) all adopted some variation of the reasonable person standard set out in Waltz and Scheunerman. Court decisions nearly unanimously consider lack of IC as a matter of negligence of the HCP to disclose necessary information to patients. Therefore, in practice the materiality test is now legal doctrine in the common law countries considered and an HCP’s failure to fulfil their fiduciary duty to disclose material risks as part of the process of IC would be considered liable in negligence.

We have briefly reviewed the evolution of the duty of disclosure, which is more commonly known as informed consent, from its origins as a paternalistic one in which the practitioner could choose what to disclose or not disclose to the patient to the current patient-centric model in which the practitioner must disclose not only treatment specific information, but also any material risks that the reasonable person in the patient’s position would want to know. Just as the elements of informed consent have evolved, so have the specifics of what constitutes disclosable information and the practitioner’s fiduciary duty of disclosure. This warrants further exploration because in this domain HCPs may find themselves at odds with current standards.

### Negligent nondisclosure

As part of the overall duty of care, HCPs must exercise reasonable skill and care. This includes the duty to disclose information to the client during the informed consent process. Consent requires disclosure of a provider’s valid basis for care. If a patient consents to a procedure and an undisclosed risk eventuates, if a different procedure is performed or went beyond or deviated significantly from that for which the consent was given, the HCP may find themselves defending an action in the tort of negligence. For a negligence action to succeed, damage (harm) must be proved [47]. In the clinical setting, harm is anything that worsens the patient’s condition including violation of patient autonomy. Thus, harm could be physical (pain, disability), emotional (harm to a patient’s dignity) or financial (paying for excessive or treatment(s) not medically indicated) and the harm can be prolonged. It is conceivable that an HCP may have a duty of care to disclose their own medical condition, financial interests or qualifications and experience if that information poses a material risk to their patient and nondisclosure could cause harm. Given this, it is plain to see that disclosure of material risks may take many forms. Therefore, full disclosure of the correct information is not only important, it is necessary.

As outlined above there is a fairly standard list of items required for ‘informed’ consent:

1. emphasizing the patient’s role in shared decision-making
2. disclosure of information
  - explaining the patient’s medical status including diagnosis and prognosis
  - describing the proposed diagnostic and therapeutic intervention, including the likelihood and effect of associated risks and benefits of the proposed action, including material risks
  - discussing alternatives to the proposed intervention, including doing nothing
3. prompting and answering patient questions related to the proposed course of action (NB. this involves probing for understanding, not simply asking ‘do you have any questions’), and
4. eliciting the patient’s preference (usually by signature). (NB. A signed form is not consent. The conversation between the clinician and the patient or carer is the true process of obtaining informed consent. The signature on the consent form is proof that the conversation took place and that the patient understood and agreed.)

The disclosure model stipulates disclosure is adequate only if “the physician’s basic thinking has been rendered transparent to the patient” [48]p.7. Rather than requiring a standardized disclosure of risks and benefits, the physician would instead be required to explain their decision-making process and the factors considered in making a recommendation. Since the late twentieth century, the list of disclosable information has gradually expanded to include areas previously not considered part of the therapeutic encounter. Materiality is expanding to include: practitioner experience, personal characteristics, health, disability, training, practice patterns, qualifications, statistics related to outcomes, disciplinary history, financial and research interests as well as an HCP’s religious or conscientious beliefs. Any of these may now be considered disclosable [23, 26, 27, 30, 49].

#### Disclosure of Practitioner’s physical condition

Is the practitioner’s physical condition a risk factor similar to success rate or post-procedure complications? The courts have considered consent cases in which plaintiffs argued the physician’s physical condition is material and thus should be disclosed. The opinions hinge on whether the physician’s condition could affect their ability to deliver health care. For example, for a practising surgeon or dentist, HIV positive status or Raynaud’s syndrome would likely be disclosable information whereas well controlled epilepsy or HIV positive status in a non-surgical physician would likely not be considered disclosable information. Alcohol abuse is considered a material risk that must be disclosed if it can be demonstrated that the abuse affects practitioner judgment or ability to perform and function. Similarly, a history of illicit drug use outside of work and while not on call may be disclosable if it can be demonstrated that the abuse affects practitioner judgment or ability to perform and function although it seems unlikely that failure to disclose such information would lead to a consent action by itself. However, if a risk eventuates, these matters would undoubtedly be raised in a subsequent negligence action both as a consent matter and a performance matter [27].

These examples focus on physical harm to the patient. Typically, a hindsight approach is taken. If the HCP’s physical condition contributed to the physical harm of the patient, it should have been disclosed as part of the consent process. However, given the expanded definition of harm, if the patient is not given information material to their declining the procedure or seeking care elsewhere, including the HCP’s physical condition, informed consent has not been obtained. To disclose or not to disclose, that is the question. Ginsberg’s considered advice in 2019 was:

The most reasonable approach would support a required disclosure if the physician’s health condition creates a realistic risk to the patient which would not exist in the absence of that condition [23]. p.73

#### Disclosure of practitioner training, qualifications and experience

Whether an HCP’s training and qualifications to perform a procedure or experience in performing a procedure can constitute a material risk and as such be disclosable information is a vexed question. On one hand the intent of practitioner registration is to ensure patient safety. Thus, when attending a registered or board-certified HCP, a patient should reasonably presume the practitioner is competent by virtue of their registration and thus practitioner experience should not be disclosable information. On the other hand, a practitioner’s experience or lack thereof in performing a particular procedure and their complication rate may be material to a reasonable patient, in which case the information becomes disclosable during consent and failure to disclose may open the HCP to an action in negligence. In the 1996 US case of *Johnson v Kokemoor* [50] for example, a patient rendered quadriplegic after surgery, brought an informed consent claim on the grounds that her physician failed to provide information material to her making an informed decision. In this case involving brain aneurysm surgery, the patient questioned the practitioner about his experience in performing the surgery. He told her he had performed the difficult procedure ‘dozens of times’ which he had not. Furthermore, he failed to provide the correct morbidity and mortality rate and failed to advise the patient the risk was significantly lower when the procedure was performed by a more experienced surgeon. The jury found that the surgeon

failed to [accurately] divulge the extent of his experience in performing this type of operation. The jury also found that a reasonable person in the plaintiff's position would have refused to consent to surgery by the defendant if she had been fully informed of its attendant risks and advantages.

The court ruled that a reasonable person in the patient’s position correctly informed of the practitioner’s lack of experience and provided with the correct mortality and morbidity statistics would likely have sought a more experienced surgeon. *Johnson v Kokemoor* was preceded by the 1988 Australian case of *Chappel v Hart* [51]. In *Chappel v Hart* in the practitioner failed to inform the patient that a remote risk was lower if the procedure was performed by a more experienced surgeon. The risk eventuated. The patient claimed that had she been made aware of this risk; she would have sought a second opinion and had the operation at the hands of a more experienced surgeon at a later date. The Court found for the patient.

Beyond saying that practitioner training, qualifications and experience will be judged disclosable on a case by case basis, there is no clear understanding to be gained from the courts. The overall impression is that practitioner training, qualifications and experience are generally not disclosable in consent but under certain circumstances these are likely to be elements of informed consent under the materiality test. Generally, if a technique is complex and the practitioner is relatively inexperienced in performing it, they likely have a legal duty to disclose their level of experience when a reasonable person would expect to be informed. Notwithstanding this, fraudulent misrepresentation of experience and qualifications will never be regarded by the courts as a positive. Paraphrasing Ginsberg’s advice: the most reasonable approach would support required disclosure if the physician’s relative inexperience creates a realistic risk to the patient which would not exist absent that condition.

#### Disclosure of practitioner research or economic interests

Broadly speaking case law and legislatures have placed the onus on an HCP to disclose information to clients when the HCP’s research or financial interests may affect treatment recommendations. This is in keeping with standard conflict of interest disclosure recommendations that exist in other areas of business and commerce. In the 1990 US case of *Moore v Regents of the University of California* [52] the California Supreme Court considered a suit against a physician who failed to disclose that he had financial incentives to develop and sell a lucrative cell line derived from the patient’s cells.

The Court concluded:

In soliciting the patient's consent, a physician has a fiduciary duty to disclose all information material to [that] patient's [informed] decision.

Accordingly, we hold that a physician who is seeking a patient's consent for a medical procedure must, in order to satisfy his fiduciary duty and to obtain the patient's informed consent, disclose personal interests unrelated to the patient's health, whether research or economic, that may affect his medical judgment.

#### Disclosure of professional disciplinary history

The courts paint a fairly clear picture here. There is an assumption that the outcome of disciplinary matters will be reflected in practice conditions on or undertakings by the provider. If the disciplinary matter related for example to record keeping, disclosure would not be expected. If, however, the disciplinary history relates to unprofessional conduct, competence and patient safety, a court could reasonably find that disclosure is required as part of consent. Indeed, some disciplinary decisions require HCPs to disclose the finding to their patients [23, 26, 27].

### Disclosure of religious, conscientious beliefs and practice patterns

When an HCP’s religious or conscientious beliefs impact their practice and treatment recommendations, transparency about those influences on medical judgment would follow informed consent doctrine. And, to the extent that some courts have recognized that physicians have a duty to disclose financial conflicts of interest as well as other personal interests that may affect clinical judgment, disclosure of religious conflicts affecting medical judgment is a natural extension of this reasoning [26]. Lastly, if a practitioner’s practice pattern places the patient under financial stress, this needs to be disclosed during the consent process thereby providing the patient with the opportunity to seek care elsewhere [53].

### The expanded duty of disclosure: implications for chiropractic

We have demonstrated that in the common law countries considered, a reasonable person in the patient’s position must be supplied with medically material facts if the consent is to be judged ‘informed’. Further, we have explored the expansion of materiality to include non-medical information such as physician characteristics etcetera. In doing so, we have confirmed that the courts recognize circumstances that may necessitate disclosure of non-medical information. How is a practitioner to decide what to disclose? Sawicki reminds us, “the common law principles of fiduciary duty and informed consent in medical practice arguably support the imposition of a duty on physicians to disclose personal commitments that impact the provision of a case” [54] p.87. Human decision-making is a finely nuanced thing. In health care, the lines between medical and non-medical interests often blur [25] which means that a blend of medical and non-medical information is likely to combine in the patient’s autonomous decision-making process.

Given this, the task is now to apply this information to the chiropractic profession. The first question we will examine is whether it is possible for a vertebral subluxation-based (VS-based) chiropractor to obtain informed consent or implement a compliant treatment plan. The second question to consider relates to the duty of disclosure, experience/qualifications and conflict of interest.

#### The duty of disclosure and vertebral subluxation-based care

By way of clarification, when discussing vertebral subluxation-based care we are referring to a practice ideology aligned with traditional Palmerian subluxation theory or a variant thereof. For adherents, reduction or removal of vertebral subluxation (VS) is theorized to improve health, quality of life, unleash human potential [55]. For these practitioners the chiropractic raison d’être is locating and removing subluxation. Establishing a diagnosis as recognized by the International Statistical Classification of Diseases and Related Health Problems (ICD) and a care plan is irrelevant. We note that chiropractic “vertebral subluxation” is not amongst the 14,000 disease codes available in the ICD-10. But for VS-based chiropractors, VS *is* the diagnosis and the care plan *is* subluxation removal. For these practitioners VS are hypothesised to be biomechanical changes within the spine resulting in clinically significant maladaptive effects on the body’s neurological and immune function [56]. They typically conduct a long-term vitalistic vertebral subluxation wellness focus style of practice [57]. This practice is a classic example of doctor-centered care which is at odds with twenty-first century patient-centered care [58, 59].

However, VS is highly contentious. Keating et al. reminded the profession that although volumes have been written about VS and the theoretical VS construct is embraced by much of the profession, “little if any substantive experimental evidence for any operational definition of the chiropractic lesion [VS] has been offered in clinical trials” [9] p.2. There exists no evidence in the literature that supports VS-based chiropractic care as a credible approach to primary prevention or early secondary prevention in general health [60]. Implying that VS has any clinical meaningfulness beyond a localized musculoskeletal disorder is an unsubstantiated claim. This explains why significant numbers of chiropractic teaching institutions have relegated VS to the history shelves [61]. Given this, is it possible for a subluxation-based practitioner to comply with practice standards? Is it possible for a patient to provide informed consent for the removal of an entity without credible evidence? We will first consider this from the perspective of the Chiropractic Board of Australia (CBA).

Chiropractic is a regulated health care profession in the countries under consideration. This means to be employed in that profession there is a requirement for a practice license or certificate from the governing regulatory body. Regulatory bodies set professional practice standards, investigate complaints about members of the profession and, where appropriate, discipline them. We will discuss the Australian practice standards because of our familiarity with them however, professional practice standards are fundamentally the same across the four common law countries considered.

Australian, UK and NZ chiropractors fall under a regulatory system regarded as approximating that of an “explicit” government regulation system. In Australia this is embodied in a single National Registration and Accreditation Scheme governing 15 registered health care professions including chiropractic. The profession’s role in formulating legislation is limited to consultation. Regulatory compliance is mandatory and there are punitive sanctions for non-compliance. There is little flexibility in interpretation and compliance requirements. The National Registration and Accreditation Scheme empowers and tasks government appointed National Boards with the primary directive to protect the public [62]. The attendant CBA Code of Conduct (Code) places standards upon the registrants that allows them the privilege of engaging in clinical practice.

The Code expresses that a program of care should be developed in a patient-centered and evidence-based context. This is evidenced by treatment being based on clinical need, tailored to the specific needs and expectations of each patient and should consider the natural history of the condition. Further it should be based on a reasonable clinical impression/diagnosis with any proposed management containing measurable outcomes (using validated measures) for monitoring care and occur within a reasonable estimated timeframe for achieving the expected benefit of care to the patient. Even the most superficial investigation of subluxation-based chiropractic leads to the conclusion that a VS-based chiropractor cannot provide *good practice* as outlined in the Code. This raises the question: can a patient provide informed consent to receive VS-based care?

The CBA’s informed consent section in its Code of Conduct is silent on the matter of the expanding scope of disclosure [referring here to practitioner characteristics]. The only information it advises is disclosable are the customary medically material facts. The Code of Conduct refers practitioners to a National Health and Medical Research Council (NHMRC) publication *General guidelines for medical practitioners in providing information to patients.* This NHMRC publication was rescinded in 2014 and has not been updated, however, it is available in the NHMRC archive. These general guidelines did not go beyond disclosing medically material facts. In fact, no consent code or statute was uncovered in any of the jurisdictions considered that did include the expanding scope of disclosure.

The CBA’s lack of current information on the expanding scope of disclosure leaves some within the chiropractic community ill-informed. Because the CBA’s guidance is silent, we need to look elsewhere for direction. Further guidance on the potential liability is available in Australian Civil Liability Laws and consumer protection law.

#### Australian civil liability Laws, duty of disclosure and peer professional opinion

Australian Civil Liability Laws encompass the common law principles governing negligence liability. Their scope is broad and applies to any claim for harm resulting from negligence in tort, contract, under statute or otherwise.

Division 5 of The Queensland Civil Liability Act (QCLA) deals specifically with the duties of a professional. While not explicitly referring to IC, it details the duty of disclosure held by a professional in relation to patient dealings. The disclosure requirements match those delineated by Waltz and Schunerman [43]. According to the QCLA, the professional breaches their duty of disclosure if they fail to provide risk related information including:

(a) information that a reasonable person in the patient’s position would, in the circumstances, require to enable the person to make a reasonably informed decision about whether to undergo the treatment or follow the advice;

(b) information that the doctor knows or ought reasonably to know the patient wants to be given before making the decision about whether to undergo the treatment or follow the advice [63]. p.17

The QCLA advises that peer professional opinion cannot serve as a defense for failing to meet the duty of disclosure if the court considers that that opinion is irrational or contrary to a written law [63]p.18. If the peer professional opinion is deemed irrational, the courts can ignore the opinion. Irrational in this context means there is no rational basis for the practice. Division 5 of the QCLA is revealed in the 1998 UK case, *Bolitho v City and Hackney Health Authority* which ruled that expert opinion must be based on logical and defensible grounds [64].

A 2018 Australian suitability to practise case serves as an example. In *Health Care Complaints Commission* (Tribunal) *v Limboro* [65] the practitioner pled guilty to 11 counts of advertising a regulated health service in a false, misleading way. This followed his criminal conviction for breaches of §133 of the Australian Health Practitioner National Law which deals with advertising. At sentencing in the criminal proceeding the Magistrate said:

the obligation is on you at every turn, to ensure that the material that is any way linked to you is as far as possible accurate, well researched, and provides an overall assessment of the good and the harm that it can do to an individual person [65].

The practitioner’s advertising advocated subluxation removal for preventing and curing cancer. Limboro’s defence consisted of his own testimony and peer professional opinion that:

He [the practitioner] did not believe, and had never believed, that chiropractic treatment is a treatment for, or preventative of, any kind of cancer. Nor did he ever inform patients that he could treat, or cure, cancer.

He [the practitioner] provides all of his patients with a standard form to read and sign which states that they understand that ‘We do not guarantee that we can prevent or cure any illness, injury or disease. The chiropractor’s purpose it to restore health through the natural flow of energy in the nervous system. This gives the body the maximum opportunity to heal itself’ [65].

In essence this is an admission of adherence to the VS ideology outlined above.

Expert reports requested by the criminal court and accepted by the Tribunal were in agreement. There is no credible evidence that:

- misalignment of the spine (VS) is a cause of any form of cancer;
- that chiropractic treatment can prevent any form of cancer; or
- that chiropractic treatment can cure or treat any form of cancer [65].

These views were also in accord with the understanding of the current state of scientific knowledge concerning the limits of chiropractic treatment held by the professional members of the Tribunal. The Tribunal also referred to a CBA statement issued on 7 March 2016 which advised registrants:

The Board is concerned about a number of practitioners who are making claims in advertising that there is a relationship between manual therapy (e.g. manipulation) for spinal problems and achieving general wellness or treating various organic diseases and infections; or that spinal problems may have a direct role in various organic diseases and infections. There is insufficient scientific evidence to support these claims.

Advertising claims that are contrary to high level evidence are unacceptable. High level evidence will usually take the form of meta-analyses, systematic reviews or one or more high quality and well respected and acknowledged studies [66].

The Tribunal proceeded on the basis that it is undisputed that chiropractic treatment is not a treatment for, or preventative of, any kind of cancer. The practitioner was found unfit to be a chiropractor and his registration was cancelled for two years.

The findings of the criminal court and the Tribunal could be regarded as a rejection of VS-based practice. A VS-based practitioner advertised that chiropractic could cure or prevent a serious organic disease, cancer. When examined, he testified that chiropractic does not treat any disease, rather it removes nerve interference thereby enhancing the body’s innate recuperative powers. The Court and the Tribunal rejected this testimony relying instead on the most accurate, well researched scientific information which concludes chiropractic care is ineffective for anything other than a well-defined list of musculoskeletal conditions. Any claim to the contrary is misleading, deceptive or likely to deceive.

#### Consumer protection Law and VS-based Care

Consumer protection laws safeguard buyers of goods and services, and the public, against unfair practices in the marketplace. Besides other provisions, these laws prohibit misleading and deceptive representations about the quality, availability, effectiveness of goods or services and a buyer’s need for the goods or services. We will discuss Australian Consumer Law because of our familiarity with it however, similar consumer laws are in place in the four common law countries considered.

The Australian Consumer Law (ACL) includes, amongst other things, a national law guaranteeing consumer rights when buying goods and services including health care services. Misleading and deceptive representations or representations likely to deceive about goods and services are breaches of the ACL. They attract penalties, enforcement remedies and consumer redress options. Subluxation-based chiropractors customarily recommend regular ongoing appointments for subluxation detection and removal. Under the ACL, representations made to people regarding any future matter must be supported by *reasonable grounds*. Opinions may be deemed misleading and deceptive if the person making the statement does not have reasonable grounds on which to base them. The Australian courts consider reasonable grounds to be clinical science validity, basic science consistency or logical reasoning, demonstrated by reproduceable published results, preferably a systematic review [67]. For example, chiropractors have reasonable grounds for claiming efficacy in managing some musculoskeletal disorders [68, 69]. In addition, under the ACL, a seller (HCP) must not make false claims about a buyer’s need for goods or services. We believe it can be argued that there is no evidence to establish the need for VS-based care for patients, therefore any claims about its purported benefits are misleading. Consequently, it would be difficult to recommend VS care and meet the expectations of the Australian Consumer Law. The ACL also prohibits bait advertising and unsubstantiated credence claims. It is beyond the scope of this paper to examine these aspects of VS care and consumer law, but as a thought experiment one is drawn to the conclusion that VS-based practitioners could have a case to answer if they advertise care for musculoskeletal disorders and then convert patients to subluxation based care claiming it to be superior to non-VS-based chiropractic.

#### The extended duty of disclosure and chiropractor experience/Qualifications & Conflict of interest

An internet search using ‘chiropractic weekend technique seminar’ yields multiple different techniques claiming that even a single weekend seminar is sufficient to begin using the technique Monday morning. We will not delve into the quality of these seminars or the claims made about the effectiveness of the techniques, we simply raise the question: Would a reasonable person in a patient’s position consider it material to be informed that their practitioner ‘learned’ the proposed technique in a seminar the weekend beforehand? Would a reasonable person be entitled to know their HCP’s reasonable grounds for providing the proposed care? Based on Court findings discussed above, it is conceivable there is a duty to disclose. Similarly, where practitioners sell products or services in their clinics, for example orthotics, pillows or radiographic services, is it conceivable that the practitioner has a duty to disclose their training to prescribe products or the availability of reasonable alternatives to the products and services offered? Clearly a conflict of interest exists between the practitioner’s business interest [selling products/services] and their fiduciary duty [placing their patient’s interest above theirs]. Court rulings would suggest there is a duty to disclose to facilitate an informed decision by the patient.

## Discussion

We have examined the evolution of the duty of disclosure and highlighted implications this brings. We now briefly consider the duties of registration boards, professional associations, and chiropractic teaching institutions should have in this domain and that individual practitioners must have.

### Chiropractic registration Boards

A Registration board’s primary duty is to safeguard the public by ensuring registrants are suitably qualified and compliant with a code of conduct, practice standards or statutes. As discussed, the CBA’s code of conduct is silent on disclosing non-medical information. The other codes we have examined are similarly deficient. Regulatory bodies must keep abreast of these changes and update their codes, standards and statutes.

### Professional associations

Professional Associations exist to support their membership. It would therefore seem logical that they would clearly guide members regarding their duty of disclosure. Continuing professional development modules would need to be updated to keep up with the evolution of informed consent and any other pertinent matters. To do anything less would be a disservice to the membership.

### Chiropractic program accreditation and VS-based practice

Chiropractic teaching programs are accredited by regulatory agencies known as Councils on Chiropractic Education (CCEs). CCEs set competencies graduates must attain before graduation and standards the chiropractic programs must meet when delivering their curricula. In this way, CCEs indirectly influence patient care and safety through their role of ensuring the standards of training delivered by chiropractic educational institutions [70]. CCEs are therefore well positioned to ensure that ethics & jurisprudence units taught at chiropractic programs are updated to keep up with the evolution of informed consent.

It would be logical to assume that CCE standards would be in step with those of the broader society. However CCEs standards have been silent on VS-based care despite it conflicting with regulatory, consumer and civil standards [71]. We note that within the same CCE regions there can be programs teaching VS-based care and chiropractic programs teaching VS only within chiropractic history units. One wonders about the implications of this for the CCEs. How can a program teaching VS-based care become accredited if their graduates are exposed to negligence in consent liability because of the ideology underpinning the program? We also muse on the information that VS chiropractic programs should disclose to prospective students for them to make an informed decision to undergo training based on an historical ideological model that does not meet societal expectations for informed consent. These questions are beyond the scope of this paper, but we suggest that the profession must grapple with them.

### Individual practitioners

The fiduciary relationship remains as it was when the concept emerged in the eighteenth century. Historically, a fiduciary relationship arises when a person (fiduciary) undertakes to act in the fiducie’s (fiducie – the person whose goodwill is held in trust by the fiduciary [72]p.243) best interests or is obliged to do so *and* the fiducie places their trust/confidences in the fiduciary to do so. This exemplifies the doctor-patient relationship. The relationship is predicated on three duties: care, loyalty and disclosure. Over time, the legal system has split these duties into separate sections of the law. What is considered in the best interest of the patient has evolved in terms of the primacy of patient autonomy and the broadened scope of disclosure.

From a legal perspective this has been important because certain consequences flow from its existence. These consequences are moral and legal obligations placed on the fiduciary over and above the other duties in contract or tort [21]. Breach of a fiduciary duty may give the fiducie legal remedy. The practitioner must uphold their fiduciary duties and, of necessity, modify their practices.

## Conclusion

Informed consent is a relatively new phenomenon. Its requirements unsurprisingly evolve as societal values and expectations change. Patient autonomy in clinical decision making is now paramount. The underlying assumption for IC doctrine is that the physician’s basic thinking be transparent to the patient to provide valid care. This necessitates more than simply explaining the risks and benefits of any proposed care. The physician must explain their decision-making process. This transparency relates to disclosure of all matters, medical and non-medical, that may influence the HCP’s delivery of that care and affect the patient’s informed decision. And the arbiter of materiality is the patient, not the practitioner. The profession’s response to the evolution of the duty of disclosure is not apparent. For example, the professional regulatory body is silent on matters of guidance beyond disclosing medically material facts. The profession needs to recognize that dereliction of the fiduciary duties may lead to professional liability, disciplinary complaints and lawsuits. It can be informed by other sources such as consumer and consent case law.

For us this raises several concerns for chiropractic. Can a patient ever provide informed consent for the removal of an entity (VS) without credible evidence / reasonable grounds? Can VS care ever meet the code of conduct standards when it lacks an evidence base and is practitioner-centered? What is the responsible educative or punitive action for CCEs, chiropractic educators and professional associations, given this knowledge? For the individual VS practitioner, it necessitates conversations about recommending a plan of care that seeks to remove a theoretical entity without quantification for diagnosis, monitoring or discharge. For others it requires disclosures of, among others, conflict of interest when recommending ‘inhouse’ products, and recency of training.

Ultimately such matters are informed and propagated by the deliberations of the courts. Because of the developments of the law, many patients are now better informed about relevant risks and the legal obligation to provide patients with meaningful advice about risks is clear. Some within the legal profession bemoan the readiness of insuring agents to settle such matters before coming under such scrutiny and providing clarity. It is our opinion that the sign of a mature, responsible profession is that it critically self-evaluate and respond in the best interests of the patient. Further, we hope that this discussion paper serves as a steppingstone for the chiropractic profession in this self-evaluation process.

## Data Availability

Not applicable.
